# Assessment of diagnostic and analytic performance of the SD Bioline Dengue Duo test for dengue virus (DENV) infections in an endemic area (Savannakhet province, Lao People's Democratic Republic)

**DOI:** 10.1371/journal.pone.0230337

**Published:** 2020-03-17

**Authors:** Jörg Blessmann, Yvonne Winkelmann, Latdamone Keoviengkhone, Vatsana Sopraseuth, Simone Kann, Jessica Hansen, Hussein El Halas, Petra Emmerich, Jonas Schmidt-Chanasit, Herbert Schmitz, Angela Mika, Christina Deschermeier

**Affiliations:** 1 Department for Infectious Disease Epidemiology, Bernhard Nocht Institute for Tropical Medicine, Hamburg, Germany; 2 Department for Infectious Disease Diagnostics, Bernhard Nocht Institute for Tropical Medicine, Hamburg, Germany; 3 Savannakhet Provincial Hospital, Savannakhet, Lao PDR; 4 Missionsärztliches Institut, Würzburg, Germany; 5 Altona Diagnostics GmbH, Hamburg, Germany; 6 Department for Virology, Bernhard Nocht Institute for Tropical Medicine, Hamburg, Germany; 7 Department of Tropical Medicine and Infectious Diseases, Center of Internal Medicine II, University of Rostock, Rostock, Germany; 8 WHO Collaborating Centre for Arbovirus and Haemorrhagic Fever Reference and Research, Bernhard Nocht Institute for Tropical Medicine, Hamburg, Germany; 9 Faculty of Mathematics, Informatics and Natural Sciences, University of Hamburg, Hamburg, Germany; New York State Department of Health, UNITED STATES

## Abstract

**Background:**

Rapid tests detecting both dengue virus (DENV) NS1 antigen and anti-DENV IgM and IgG antibodies facilitate diagnosis of dengue fever (DF) in resource-poor settings.

**Methodology/principal findings:**

92 acute phase serum samples from patients with a PCR-confirmed DENV infection collected in Lao People’s Democratic Republic (Lao PDR) in 2013 and 2015 were analyzed with the SD Bioline Dengue Duo test. A subset of 74 samples was additionally tested with the Platelia NS1 antigen test, the Panbio DENV μ-capture ELISA and the Panbio DENV IgG ELISA. IgM seroconversion was assayed using follow-up samples of 35 patients collected in the convalescent phase. 57.6%, 22.8% and 44.6% of acute phase serum samples tested positive in the SD Bioline Dengue Duo NS1, IgM, and IgG test, respectively. Diagnostic sensitivity of the SD Bioline Dengue Duo NS1 test strongly correlated with viral load, decreased rapidly over the acute phase of the disease, and was significantly reduced in presence of high anti-DENV IgG antibody titers resulting from secondary DENV infection. While a good concordance (Cohen’s kappa 0.78) was found between the SD Bioline Dengue Duo NS1 test and the Platelia NS1 antigen ELISA, both the SD Bioline Dengue Duo IgM and IgG test displayed a significantly lower sensitivity than the corresponding ELISA tests.

**Conclusions/significance:**

The SD Bioline Dengue Duo test is a valuable tool for diagnosis of DENV infections especially when analyzing early acute phase samples with high viral load. Nevertheless, in endemic areas, where secondary flavivirus infections are common, diagnostic sensitivity of the NS1 and IgM test components may be compromised.

## Introduction

With an estimated incidence of 100 million clinically apparent cases per year in Asia, Latin America and Africa, dengue fever is the most frequent arboviral disease globally [1]. In endemic countries, disease burden has increased dramatically over the recent years due to population growth and urbanization. Globalization and climate change facilitate the geographic spread of both the mosquito vector and the pathogen to previously unaffected areas [1].

Transmission of the four genetically distinct DENV serotypes mainly occurs by the bite of infected *Aedes aegypti* mosquitoes, limited vectorial capacity of *Aedes albopictus* has also been shown [2]. As in other flavivirus infections, asymptomatic or sub-clinical infection is frequent. In an estimated 25% of infected individuals, a sudden onset of high-grade fever occurs after an incubation period of 4–7 days, often accompanied by other unspecific disease symptoms like headache, myalgia, arthralgia, nausea, vomiting, and rash. During this febrile phase, leucopenia and thrombocytopenia are frequently observed; this finding (as the occurrence of petechiae and/or a positive tourniquet test) can support differentiation of DF from other febrile illnesses [3]. Upon defeverescence, usually occurring between days 3–7 of illness, most patients start to recover from the disease. Nevertheless, some patients develop severe symptoms caused by increased vascular permeability, plasma leakage and intravascular volume depletion that can be lethal if left untreated.

Historically, cases have been classified according to WHO guidelines in the categories “dengue fever” and “dengue hemorrhagic fever”, the latter including also the most severe grade, the dengue shock syndrome. In an attempt to adapt the system better to the clinical situation [4,5] this classification has been revised in 2009 and is since then replaced by the categories “dengue infection with warning signs”, “dengue infection without warning signs” and “severe dengue infection” [6].

Laboratory diagnostics of DENV infection can be based on the detection of the virus itself (by virus culture), viral RNA (by real-time PCR), viral antigen (by ELISA or lateral flow testing) and the detection of a virus specific IgM/IgG response [1,6]. Thereby, direct methods (virus culture, real-time PCR, antigen detection) as well as (with limitations) IgM detection can be used for diagnosis of acute infections while the presence of DENV-specific IgG antibodies indicates a prior exposure to DENV [6]. Although infection with one DENV serotype confers lifelong homotypic immunity, heterotypic cross-protection is timely limited [1]. Thus, consecutive infections with different serotypes are commonly observed in residents of endemic countries. Serologically, these secondary infections are characterized by the rapid and early rise of broadly reactive anti-flavivirus IgG antibodies; IgM antibody titers often remain low [6].

Dependent on local disease epidemiology, travel history and clinical signs, differential diagnosis of DF against a wide range of diseases caused by bacterial (e.g. spotted fever, scrub typhus, leptospirosis), parasitic (e.g. malaria) or viral (e.g. chikungunya fever, Zika fever, influenza, measles) pathogens is necessary [1,6]. Thereby, an accurate diagnosis already in the early febrile phase of the disease is important to (1) avoid further unnecessary and expensive diagnostic procedures and potentially harmful antibiotic treatments, (2) administer correct and timely interventions already at primary and secondary care levels and (3) to identify outbreaks as early as possible. Thus, time-consuming, highly specialized methods as virus cultivation or serological evaluation based on the observation of seroconversion in paired serum samples is of little relevance in the acute clinical situation and the most relevant techniques for early diagnosis in the field are DENV-specific real-time PCR and the detection of virus antigen by rapid test or ELISA.

In resource-poor settings, lateral flow based point-of-care tests (as the SD Bioline Dengue Duo test) may be the only option to investigate those parameters because they do not require expensive technical equipment and are not affected by adverse storage temperature conditions [7]. Here, we assess the analytical and diagnostic sensitivity of this rapid test using sera from patients with a PCR-confirmed DENV infection from an endemic region in Southeast Asia (Savannakhet province, Lao PDR) [8,9].

## Methods

### Ethics statement

The study complies with the Declaration of Helsinki. Written informed consent was obtained from all individuals or, in case of minors, from parents or legal guardians before enrollment. Data privacy protection was guaranteed by anonymization of serum samples. Collection of serum samples was approved by the Ethics Committees of the Lao People’s Democratic Republic (No. 030/NECHR), the Hospital Rosario Pumarejo de Lopez of Valledupar/Colombia, and the Medical Association Hamburg (No. PV4608).

### Collection and on-site analysis of sera

Sera of DF patients were collected during the studies „Diagnosis of causative agents in Lao patients with undifferentiated fever and acute encephalitis syndrome”at the Savannakhet Provincial Hospital (Savannakhet/Lao PDR, 2013–2015) and „Diagnosis of patients with febrile infections”at the Hospital Rosario Pumarejo de Lopez (Valledupar/Colombia, February–August 2014). Routine diagnostic testing (FTD tropical fever core multiplex real-time PCR (FastTrack Diagnostics) and/or SD Bioline Dengue Duo test (NS1, IgM, IgG) (Alere/Abott)) of acute phase samples was performed on-site right after hospital admission according to the manufacturers’ instructions. Residual samples were stored at -20°C and shipped on dry ice to BNITM (Hamburg/Germany) for further analysis.

### DENV diagnostic testing

In addition to the on-site analysis, the following diagnostic tests were performed according to the manufacturers’ instructions: RealStar**®** Dengue RT-PCR Kit 1.0 (Altona Diagnostics), RealStar**®** Dengue Type RT-PCR Kit 1.0 (Altona Diagnostics), PLATELIA**™** Dengue NS1 Ag ELISA (Bio-Rad), Panbio**®** Dengue IgM Capture ELISA (Alere/Abbott), Panbio**®** Dengue IgG Indirect ELISA (Alere/Abbott). For the DENV IgG indirect immunofluorescence test (IIFT), Vero cells infected with DENV serotype 2 were plated on glass slides and fixed with aceton. After blocking, glass slides were incubated with patient sera diluted in PBS and incubated for 1 h at 37°C. After washing with PBS, glass slides were incubated for 20 min at 37°C with secondary antibody (FITC labeled anti-human IgG (Sifin Diagnostics GmbH), 1:350 in PBS) and washed again with PBS before microscopic examination.

### DF patient samples included in the analysis

For details, see STARD chart in the Supporting Information. Briefly, 316 and 454 patients with febrile diseases were admitted to the Savannakhet Provincial Hospital and the Hospital Rosario Pumarejo de Lopez of Valledupar, respectively, during the respective study periods. For 120/316 (38%) of Lao PDR patients and 35/454 (8%) of Colombia patients, an acute DENV infection was confirmed by the RealStar**®** Dengue RT-PCR Kit 1.0 (Altona Diagnostics, Hamburg, Germany). For 92 and 26 of these patients, respectively, a complete dataset comprising conclusive PCR-based serotyping, SD Bioline Dengue Duo Rapid test results and DENV IgG IIFT results was available; only samples from those patients were included in the analysis.

### SD Bioline Dengue Duo NS1 test using samples after immune complex dissociation

Acid treatment was performed as described by Lapphra *et al*. [10]. Briefly, 50 μl serum were mixed with 50 μl 1.5 M glycine pH 2.8 and incubated for 1 h at 37°C before adding 10 μl 1.5 M Tris pH 9.4. SD Bioline NS1 antigen testing was performed immediately afterwards by applying 100 μl of the reaction mixture to the sample well. Heat treatment was performed as described by Beall *et al*. [11]. Briefly, 70 μl serum were mixed with 70 μl 0.1 M EDTA pH 7.5 and incubated for 5 min at 95°C. Following centrifugation (12,000 g, 10 min), 100 μl of the supernatant was applied to the sample well of the SD Bioline Dengue Duo NS1 test. For comparison, 50 μl serum were diluted with 60 μl 1 x PBS pH 7.4 and 100 μl of this mixture were applied to the sample well of the SD Bioline Dengue Duo NS1 test. According to the manufacturer’s instruction, all test results were monitored after 20 min incubation at room temperature.

### Data analysis

Statistical testing was performed using GraphPad QuickCalcs. For categorical and continuous data, Fisher’s exact test and Student’s t test was used, respectively. Concordance of diagnostic tests was quantified by calculation of % agreement and Cohen’s kappa [12].

## Results

### Diagnostic sensitivity of the SD Bioline Dengue Duo NS1 antigen test depends on viral load and decreases rapidly over the acute phase of the disease

**Fig 1:**
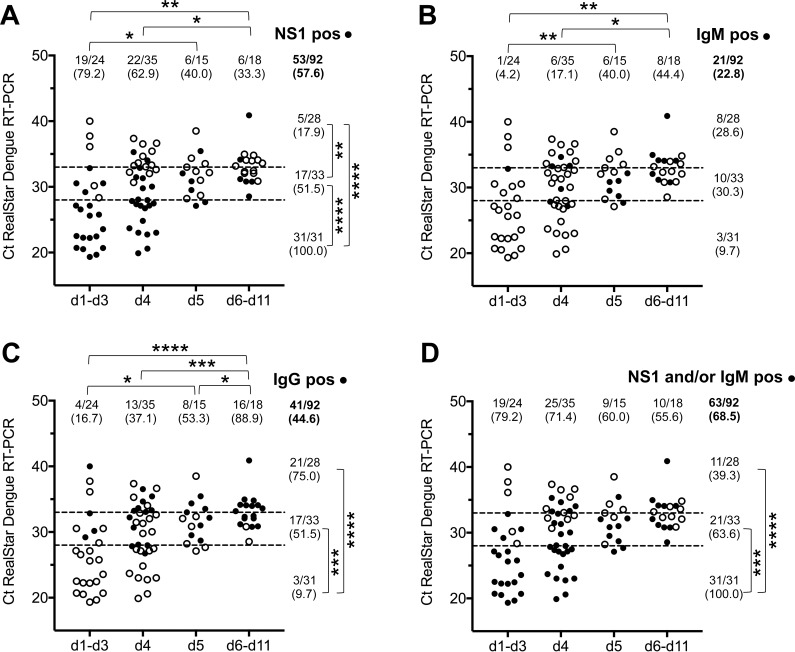
SD Bioline Dengue Duo test results. Acute phase serum samples from 92 patients with a PCR-confirmed DENV infection were analyzed with the SD Bioline Dengue Duo NS1 **(A,D)**, IgM **(B, D)** and IgG test **(C).** Samples were stratified according to sampling day d (post onset of symptoms) and Ct-value (categories low (≤ 28.0), medium (28.0 < Ct ≤ 33.0), high (Ct > 33.0) indicated by dashed lines). Open/filled circles represent negative/positive results. Absolut sample numbers (n positives / N samples in category) and percentages are displayed. Statistically significant differences are indicated (*: p < 0.05, **: p < 0.01, ***: p < 0.001, ****: p < 0.0001).

Acute phase serum samples (median 4 days post onset of symptoms) from 92 Lao patients (**Table 1**) with a PCR-confirmed DENV infection were analyzed using the SD Bioline Dengue Duo rapid test (**Fig 1**). Overall, 53/92 samples (57.6%) generated a positive result in the NS1 antigen rapid test. NS1 positivity strongly correlated with high viral load (indicated by low Ct-values) and significantly decreased from 79.2% for samples collected between day 1 and day 3 post onset of symptoms to 33.3% between day 6 and 11 (**Fig 1A**). DENV-specific IgM antibodies were detected in 21/92 samples (22.8%) with the highest percentage (44.4%) of positivity found in the samples collected later than day 5 after onset of symptoms (**Fig 1B**). In 44.6% of all samples (41/92), DENV-specific IgG antibodies were detected (**Fig 1C**) indicating a high incidence of secondary DENV infections. Overall, 68.5% of samples tested positive in the NS1 and/or the IgM test (**Fig 1D**). Similar observations were made when analyzing samples from a smaller collective (n = 26) of DF patients with a PCR-confirmed DENV infection collected 2014 in an endemic region in South America (Valledupar/Colombia [13–15]) (**S1 Fig**).

**Table 1:** Demographic and clinical parameters of DF patients (Lao PDR (2013–2015)).

patients, n	92
**age in years, median (range)**	21 (15–55)
**m/f gender, n (%)**	49/43 (53.3/46.7)
**days post onset, median (range)**	4 (1–11)
**Ct pan-DENV PCR, median (range)**	30.8 (19.3–40.9)
**DENV1/2/3/4 serotype, n (%)**	2/28/50/12 (2.2/30.4/54.3/13.0)
**WBC in 10**^**3**^ μ**l**^**-1**^**, median (range)**	2.9 (1.0–12.0)
**WBC < reference, n (%)**	66 (71.7)
**PLT in 10**^**3**^ μ**l**^**-1**^**, median (range)**	107.0 (2.0–411.0)
**PLT < reference, n (%)**	67 (72.8)

m: male, f: female, WBC: white blood cells, PLT: platelets, Ct: threshold cycle; reference values are 4.0–11.0 x 10^3^ per μl for WBC and 150.0–450.0 x 10^3^ per μl for PLT.

### Diagnostic sensitivity of the SD Bioline Dengue Duo test does not differ between DENV serotypes 2 and 3 and does not correlate with WBC and PLT counts

No correlation was observed between the main two DENV serotypes represented in the patient collective (DENV2 and DENV3) and the diagnostic sensitivity of either component of the SD Bioline Dengue test (**S2 Fig**). Furthermore, the percentage of SD Bioline Dengue Duo NS1 positivity did not differ significantly between patients with WBC and PLT counts above and below the reference values, respectively (**S3 Fig**).

### Diagnostic sensitivity of the SD Bioline Dengue Duo NS1 rapid test depends on anti-DENV IgG status

**Fig 2:**
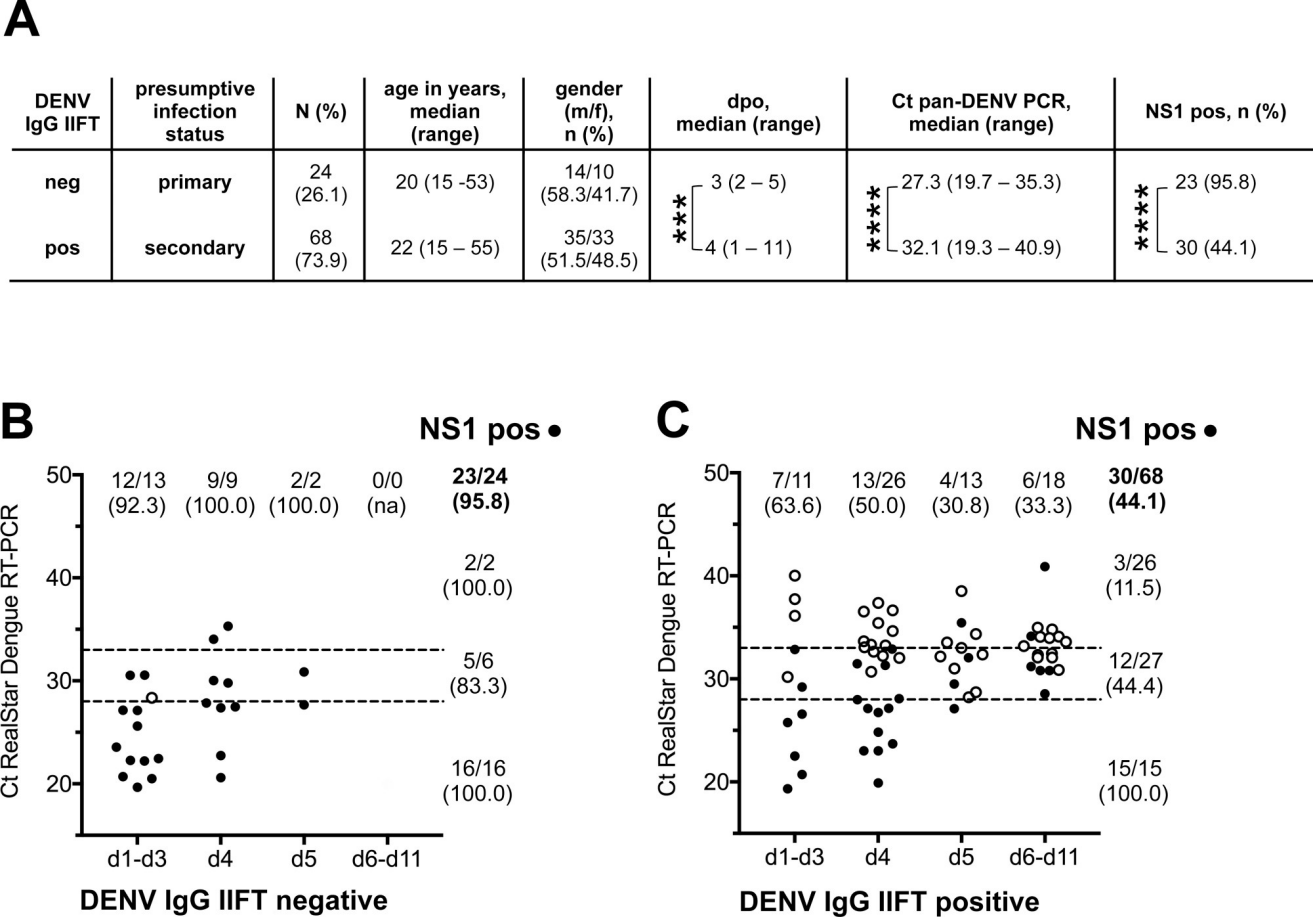
SD Bioline Dengue Duo NS1 test results stratified according to anti-DENV IgG status. Acute phase serum samples from 92 patients with a PCR-confirmed DENV infection were categorized according to their anti-DENV IgG status as defined by DENV IgG IIFT. **(A)** Tabular representation of subgroup characteristics. Ct: threshold cycle, dpo: days post onset of symptoms. **(B)** DENV IgG IIFT negative samples (N = 24), **(C)** DENV IgG IIFT positive samples (N = 68). Samples were stratified according to sampling day d (post onset of symptoms) and Ct-value (categories low (≤ 28.0), medium (28.0 < Ct ≤ 33.0), high (Ct > 33.0) indicated by dashed lines). Open/filled circles represent negative/positive NS1 test results. Absolut sample numbers (n positives / N samples in category) and percentages are displayed. ***: p < 0.001, ****: p < 0.0001.

To investigate the influence of the presence of DENV-specific IgG antibodies on the diagnostic sensitivity of the NS1 antigen rapid test, samples were categorized according to the results of the BNITM in-house DENV IgG IIFT (**Fig 2A**). While NS1 antigen was detected by the SD Bioline Dengue Duo NS1 antigen rapid test in 95.8% of the 24 DENV IgG IIFT negative samples (**Fig 2B**), only 44.1% of the 68 DENV IgG IIFT positive samples (**Fig 2C**) generated a positive result in this test.

To eliminate the direct influence of sampling day and viral load (which differ significantly between those subsets; **Fig 2A**), analysis was restricted to samples collected on day 4 post onset of symptoms. Ct values were similar for the DENV IgG IIFT negative and positive samples in this subgroup (p = 0.2892), nevertheless, all 9 DENV IgG IIFT negative samples were tested positive in the SD Bioline Dengue Duo NS1 rapid test (**Fig 2B**) while only 50% (13/26) of DENV IgG IIFT positive samples generated a positive result in this test (**Fig 2C**). Although this observation is of borderline statistical significance (p = 0.0131) due to the small sample numbers, it may indicate a direct influence of DENV-specific IgG antibodies on NS1 antigen test sensitivity.

Previously, immune complex dissociation methods have been described to enhance the sensitivity of antigen detection in dot blot [16] and ELISA applications [10,11,17–23]. Nevertheless, the published methods, employing either acid [10,11,16,19–21,23] or heat treatment [11,17,19,22] of serum samples, were found to be incompatible with the SD Bioline Dengue Duo NS1 antigen test (**S4 Fig**).

### Comparison of rapid test results with commercial ELISA tests (Platelia NS1 antigen test, Panbio μ-capture ELISA, Panbio IgG ELISA) and in-house DENV IgG IIFT

**Fig 3:**
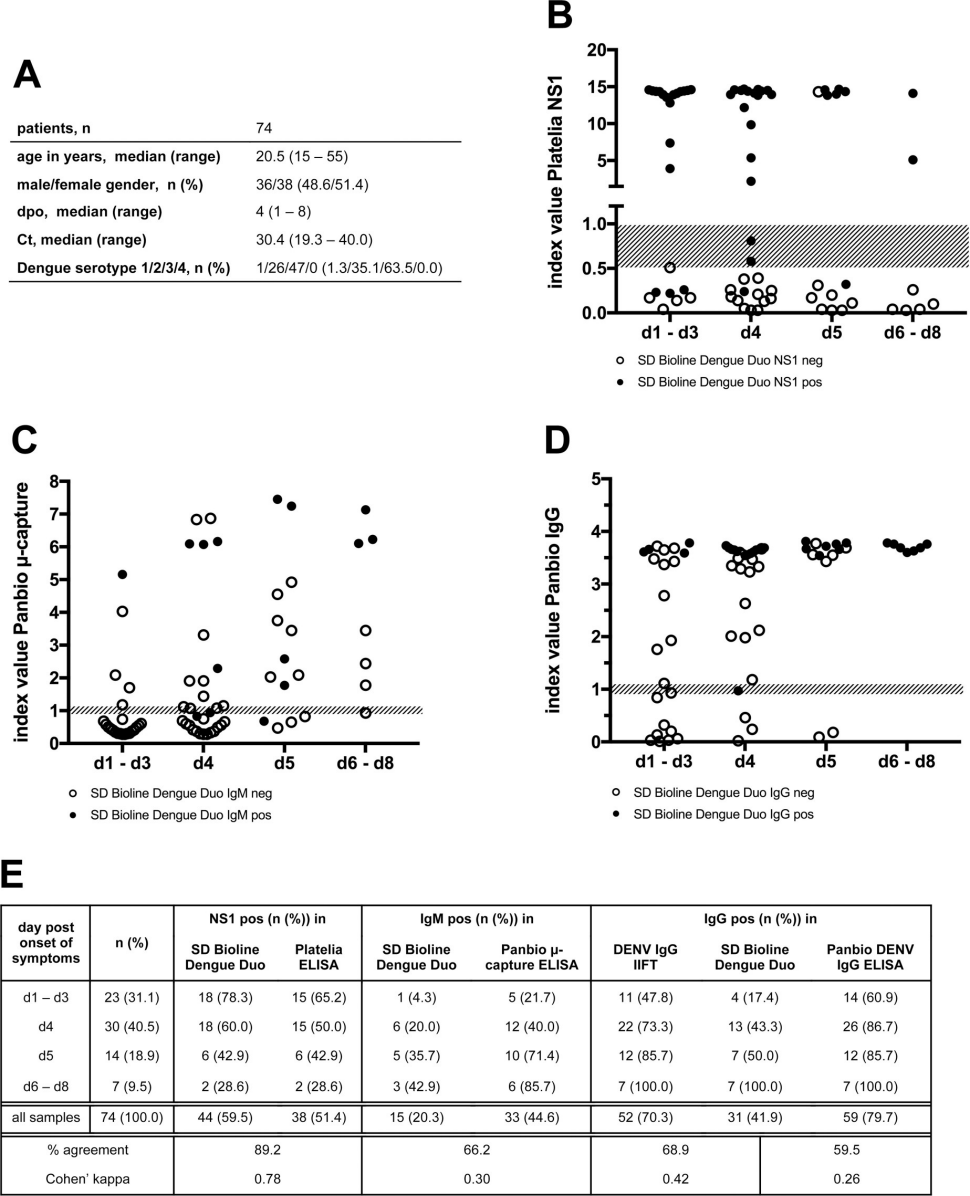
Comparison of SD Bioline Dengue Duo test results with ELISA results and DENV IgG IIFT. A subset of 74 acute phase serum samples **(A)** was analyzed with the Platelia NS1 Antigen ELISA **(B)**, the Panbio DENV μ-capture ELISA **(C)** and the Panbio DENV IgG ELISA **(D)**. Open/filled circles represent negative/positive results obtained with the corresponding SD Bioline Dengue Duo tests. Shaded areas represent index values rated as equivocal according to the respective manufacturer’s recommendations. **(E)** Tabular summary of results and statistical testing.

To compare the performance of the SD Bioline Dengue Duo test with conventional ELISA testing, 74 of the 92 acute phase serum samples (**Fig 3A**) were analyzed with the Platelia NS1 Antigen ELISA (**Fig 3B**), the Panbio μ-capture ELISA (**Fig 3C**), and the Panbio IgG ELISA (**Fig 3D**). While a good concordance was observed between the SD Bioline Dengue Duo NS1 test and the Platelia NS1 Antigen ELISA results (**Fig 3B** and **3E**; Cohen’s kappa 0.78), only a low percentage of the samples classified as IgM/IgG positive by the Panbio μ-capture and IgG ELISA test, respectively, also generated a positive result in the SD Bioline Dengue Duo IgM/IgG test (**Fig 3C and 3D**). Similarly, only a moderate agreement (Cohen’s kappa 0.42) was found between the SD Bioline Dengue Duo IgG test and the DENV IgG IIFT (Fig 3E) while the DENV IgG IIFT and the Panbio IgG ELISA were in good concordance (**S5 Fig**, Cohen’s kappa 0.75).

### IgM seroconversion in DF patients as analyzed by SD Bioline Dengue Duo IgM rapid test and Panbio μ-capture ELISA

**Fig 4:**
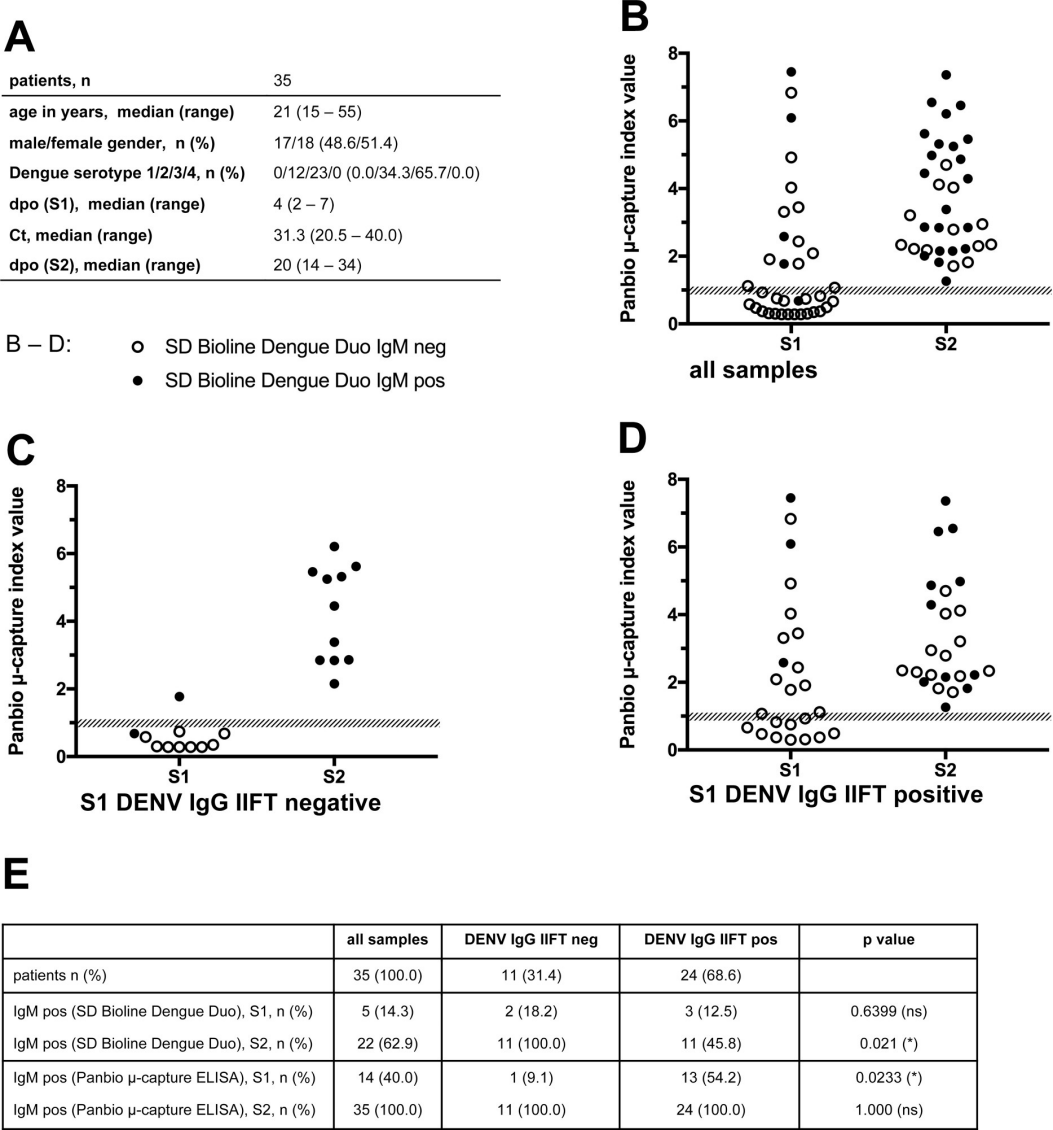
Seroconversion in DF patients detected by SD Bioline Dengue Duo IgM test and Panbio μ-capture ELISA. For 35 patients **(A)** both an acute phase serum sample (S1) and a follow-up sample taken during the convalescent phase (S2) were analyzed **(B).** Open/filled circles represent samples tested negative/positive in the SD Bioline Dengue Duo IgM test. Shaded areas represent index values rated as equivocal according to the respective manufacturer’s recommendations. **(C)** Subset of sample pairs with a DENV IgG IIFT negative S1 sample, (**D)** Subset of sample pairs with a DENV IgG IIFT positive S1 sample. **(E)** Tabular summary of results. *: p < 0.05, ns: not significant.

For a subset of 35 patients (**Fig 4A**), a follow-up sample (“S2”) could be obtained in the convalescent phase of the disease (median day 20 post onset of symptoms). While all S2 samples were tested positive in the Panbio IgM ELISA, only 22/35 (62.9%) of samples were detected as DENV IgM positive in the SD Bioline Dengue Duo IgM test (**Fig 4B**). A significant difference was observed between patients with a different anti-DENV IgG status of S1 samples (**Fig 4E**): IgM antibodies were detectable in the S2 sample by the SD Bioline Dengue Duo IgM test in 11/11 (100.0%) of patients with an DENV IgG IIFT negative S1 sample (**Fig 4C**), but only in 11/24 (45.8%) of patients with an DENV IgG IIFT IgG positive S1 sample (**Fig 4D**).

### Specificity of the NS1 and IgM tests

To assess assay specificity, we tested 173 sera from Laotian patients with febrile disease admitted to Savannakhet Provincial Hospital in 2013–2015 (**Table 2, S6 Fig).** The SD Bioline Dengue Duo NS1/IgM tests and the Platelia NS1 ELISA showed excellent specificity of > 96%. The Panbio μ-capture ELISA detected isolated IgM in 6 (12.8%) of 47 DENV RT-PCR negative patients not classified as presumptive DF.

**Table 2:** Specificity of SD Bioline Dengue Duo NS1 (NS1 RT), Platelia NS1 Antigen ELISA (NS1 ELISA), SD Bioline Dengue Duo IgM (IgM RT), and Panbio DENV μ-capture ELISA (IgM ELISA).

category	N	n (%) pos in			
		NS1 RT	NS1 ELISA	IgM RT	IgM ELISA
**Malaria**	22	0 (0.0)	nt	0 (0.0)	nt
**Chikungunya fever**	1	0 (0.0)	nt	0 (0.0)	nt
**Rickettsia infection**	2	0 (0.0)	nt	0 (0.0)	nt
**Tsutsugamushi fever**	10	0 (0.0)	nt	0 (0.0)	nt
other febrile disease* **(DENV RT PCR neg)**	52	1 (1.9)	2 (3.8)	3 (5.8)	9 (17.3)
other febrile disease** **(DENV RT PCR nt)**	86	1 (1.2)	nt	1 (1.2)	nt
all samples (n/N (%))		2/173 (1.2)	2/52 (3.8)	4/173 (2.3)	9/52 (17.3)

* including 5 presumptive DF cases

** including 2 presumptive DF cases; nt: not tested

## Discussion

In this work, we describe the diagnostic performance of the SD Bioline Dengue Duo rapid test in comparison to commercial ELISA kits (PLATELIA**™** Dengue NS1 Ag ELISA, Panbio**®** Dengue IgM Capture ELISA, Panbio**®** Dengue IgG Indirect ELISA) and an in-house DENV IgG IIFT in an endemic setting (Savannakhet province, Lao PDR).

Patient sera were collected between 2013 and 2015 at Savannakhet Provincial Hospital in southern Lao PDR. On-site routine diagnostic testing was performed for all febrile patients enrolled in the study in 2013 using the FTD tropical fever core multiplex real-time PCR kit simultaneously testing for dengue virus, chikungunya virus, west nile virus, *Plasmodium* spp., *Rickettsia* spp., *Leptospira* spp., and *Salmonella* spp.. Although co-circulation/co-infection of dengue and chikungunya virus was detected in samples originating from Champasak province during the 2013 DF outbreak in southern Lao PDR [9], no co-infections with CHIKV were observed in the DF patients enrolled in our study. During the 2013 study period, only one out of 172 febrile patients admitted to Savannakhet Provincial Hospital was found to be CHIKV PCR-positive. Interestingly, this patient reported to have recently returned from a travel to Champasak province. Collected before the first autochthonous ZIKV case in Lao PDR had been reported to the WHO in 2016 [24], samples were not tested on-site for ZIKV (co-)infection. A retrospective analysis of acute phase serum samples archived in 2012–2013 from Laotian patients with suspected dengue fever revealed the presence of ZIKV in 18 (1.3%) of 1353 tested samples [24]. Thus, ZIKV (co-)infections cannot be ruled out completely, but their abundance is presumably low.

With percentages ranging between 37% and 75%, DENV1 had been the predominant serotype detected in DF patients in Lao PDR over several years (2007–2011) [25]. This changed significantly in 2012, when DENV3 was identified as the causing pathogen in 94% of patients [25]. In the following year 2013 (when the majority of patients for the study presented in this work were recruited), the by far predominant serotypes found in DF patients admitted to Savannakhet Provincial Hospital were DENV2 and DENV3. An infection with DENV1 was found only in two out of 99 patients with an assignable DENV serotype and no DENV4 cases were seen in 2013. Similarly, only DENV2 and DENV3 serotypes were detected during the 2013 DF outbreak in Champasak province (southern Laos) [9]. In the later phase of the study (2015), only sporadic DF cases were seen at the Savannakhet Provincial Hospital (N = 18) with DENV4 being the predominant serotype (N = 12).

Analysis of 92 acute phase samples (median day 4 post onset of symptoms) from patients with a PCR-confirmed DENV infection revealed an overall diagnostic sensitivity of the SD Bioline Dengue Duo NS1 test of 57.6%. NS1 positivity strongly correlated with viral load (indicated by RT-PCR Ct-value) and decreased rapidly over the acute phase of the disease as had been observed previously on patient collectives from Vietnam [26,27] and Colombia [28]. In contrast, no significant change in diagnostic sensitivity between days 2 and 7 after onset of symptoms had been observed recently in a study employing samples from Brazil [29].

IgG antibodies recognizing DENV antigens were detectable by the in-house DENV IgG IIFT in 74% (68/92) of acute phase serum samples (**Fig 2**) indicating a high percentage of secondary DENV infections in the Lao PDR study collective [6]. In concordance with previous studies analyzing samples from Asia [26,27,30] and the Americas [30], we observed a significantly reduced diagnostic sensitivity of the SD Bioline Dengue Duo NS1 test in secondary DENV infections. By stratifying the samples according to viral load and sampling day, a direct influence of the presence of DENV-specific IgG antibodies on the diagnostic sensitivity of the SD Bioline Dengue Duo NS1 test could be shown. Thus, the low diagnostic sensitivity of NS1 antigen testing in patients of our study collective with a detectable DENV IgG antibody titer in the acute phase sample is most likely caused by both a reduced viral load leading to lower NS1 serum levels and by the formation of NS1/IgG immune complexes that are not detectable due to masking of epitopes and/or sterical hindrance [26,31].

Immune complex formation between circulating antigens and antigen-specific antibodies has already been recognized as a factor negatively influencing diagnostic antigen detection in a variety of different disease conditions including HIV infection [20–22], West Nile fever [18], and Dengue fever [10,16,17,19,23]. Nevertheless, the DENV IgG assays applied in this work either employ native virus particles displaying all antigens (DENV IgG IIFT) or a recombinant DENV envelope protein E (SD Bioline Dengue Duo IgG rapid test); the antigen used in the Panbio DENV Indirect IgG ELISA has not been specified in the instructions for use. Therefore, these test results do not allow conclusions about the anti-NS1 IgG titers in the respective samples. Indeed, the anti-DENV IgG response is largely dominated by antibodies against structural antigens E and prM [32]. Anti-NS1 IgG titers may vary significantly between patients from different endemic areas [16] and both anti-NS1 titers and recognized epitopes correlate with disease severity [33].

A conclusive analysis of the influence of anti-NS1 IgG antibodies on NS1 antigen detection in our patient collective as well as the sound implementation and validation of an immune complex dissociation method for improvement of NS1 rapid test sensitivity would require: 1) Determination of anti-NS1 antibody titers in all tested samples by ELISA or IIFT. 2) Implementation of an immune complex dissociation method efficiently disintegrating immune complexes but preserving NS1 protein structure/epitopes as necessary for the respective antigen detection method. 3) Establishment of a neutralization control confirming signal specificity in post-dissociation samples: as discussed by Pokriefka *et al*. [21], positive test results should be suppressible by the addition of an excess of an anti-antigen positive serum due to reformation of immune complexes.

Common methods for *in vitro* immune complex dissociation are acid treatment (using either HCl [20,21] or glycine [10,16,19,21,23], heat exposure [17,19,22], and alkaline treatment [18]. Although all of these methods have been reported to potentially increase accessibility of flavivirus NS1 epitopes in Western Blot and/or ELISA applications [10,16,18,19,23], evidence is strong that these relatively harsh treatments can also prevent efficient antigen detection in some samples. For example, Koraka *et al*. observed a loss of signal in 8/32 (25%) of pre-dissociation NS1 positive samples in a dot blot application [16]. Similarly, acid treatment led to an overall increase of the diagnostic sensitivity of an DENV NS1 antigen capture ELISA developed by Shen *et al*. [23], but significantly reduced P/N ratios [23].

In a pilot experiment employing selected NS1 positive serum samples NS1 detection by the SD Bioline NS1 antigen rapid test was completely abolished by standard glycine [10,16,19] and heat treatment [17,19] (**S4 Fig**). Several reasons can account for this apparent methodical incompatibility: 1) Acid treatment may (partially) denature DENV NS1, preventing efficient binding of monoclonal antibodies recognizing conformational epitopes and thereby hamper efficient binding to the gold-labeled mouse anti-NS1 antibody and/or the detection antibody on the test line. 2) Even if binding to the gold-labeled anti-NS1 antibody to the acid-treated NS1 protein is still possible, efficient flow of the formed complexes across the membrane strip may be hindered by the denaturation-induced formation of larger protein aggregates. 3) The buffer conditions in the acid treated/neutralized samples may interfere with binding of the NS1 protein to the gold-labeled antibody or the antibody on the test line. Thus, alternative IC dissociation methods employing milder conditions, e.g. 0.1 M glycine pH 2.8 (as is commonly employed for elution of proteins from immunoaffinity columns) and shorter exposure times to dissociating conditions [34], should be tested for their compatibility with NS1 antigen detection assays. An elegant alternative approach to enable NS1 antigen detection in samples from patients with secondary infections in an ELISA format would be not to aim for permanent, complete dissociation of serum ICs but to exploit their presence/reformation after a short term, mild dissociation by directly detecting them using an immune complex receptor molecule like C1q, CD16, or CD32 [35] as capture molecule.

No significant difference was observed between the diagnostic sensitivities of the SD Bioline Dengue Duo NS1 for the two main DENV serotypes (DENV2, DENV3) represented in our study collective. In contrast, Hang *et al*. observed a significantly lower diagnostic sensitivity of both the PLATELIA**™** Dengue NS1 Ag ELISA and an NS1 lateral flow rapid test in DENV2 than in DENV3 samples from Vietnam [26]; nevertheless the observed result could also have been caused by differences in both viral load and serological status of the DENV2 and DENV3 samples, respectively [26]. A lower diagnostic sensitivity of the SD Bioline Dengue Duo NS1 test for DENV2 and DENV4 in comparison to DENV1 and DENV3 had also been reported in a Colombian study [28].

Decreased white blood cell and platelet counts are more frequently observed in DENV infection than in other febrile diseases [3]. While an inverse correlation between NS1 antigen levels and the WBC nadir had been described in Sri Lankan patients [36], no significant difference in SD Bioline Dengue Duo NS1 positivity was detected between acute phase patient samples with WBC and PLT counts above and below the respective lower reference values in our study.

The SD Bioline Dengue Duo rapid test results for NS1 antigen and IgM/IgG antibodies were compared to corresponding commercially available ELISA tests. As was reported previously for a sample panel originating from French Guiana [37], the SD Bioline Dengue Duo NS1 test results were in good concordance with the PLATELIA**™** Dengue NS1 Ag ELISA. In contrast, significantly fewer samples were classified as positive in the SD Bioline Dengue Duo IgM test than in the Panbio**®** Dengue IgM Capture ELISA, resulting in low Cohen’s kappa value of 0.3. Similarly, an only moderate agreement (Cohen’s kappa = 0.53) was found between the SD Bioline Dengue Duo IgM test and another commercial DENV IgM ELISA (Dengue Fever IgM Capture DxSelect**™,** Focus Diagnostics) by Simonnet *et al*. [37]. The analytical sensitivity of the SD Bioline Dengue Duo IgM test was found to be particularly low in comparison to the Panbio DENV IgM capture ELISA when serum samples from patients with high anti-DENV IgG titers were analyzed. Thus, the difference may be caused by a potential supplementation of the ELISA kit’s sample diluent with an IgG pre-absorbing reagent not explicitly commented on in the Instructions For Use.

The SD Bioline Dengue Duo IgG test preferentially detected samples with a DENV IgG IIFT titer > 1:2,000 (**Fig 3, S5 Fig**) Therefore, a positive result of the SD Bioline Dengue Duo IgG test in an acute phase sample is strongly indicative of a secondary DENV infection. Although a good agreement (Cohen’s kappa = 0.75) was found between the in-house DENV IgG IIFT and the Panbio DENV IgG ELISA, seven samples generating a weak to medium high signal in the ELISA were classified as DENV IgG negative by the in-house DENV IgG IIFT. This divergence may either be caused by false positive ELISA results or a reduced capacity of the DENV2-based IgG IIFT to detect low levels of IgG antibodies resulting from a previous infection with a different serotype. Notably, an optical density at the upper limit of the detection range was obtained for the majority of tested samples with the Panbio DENV IgG ELISA indicating a saturation of the obtained signals. Therefore, the commonly used categorization of samples according to IgM/IgG index ratios to analyze the patients’ infection status (primary vs. secondary infection) [33,38,39] is not applicable to our dataset.

As already reported by previous studies including samples from patients infected with different viral, bacterial and parasitic pathogens [26,28,29,40,41], specificity was found to be high (> 97%) for both the SD Bioline Dengue Duo NS1 and the SD Bioline Dengue Duo IgM test when testing samples from Laotian patients suffering from other febrile diseases including malaria, chikungunya, scrub typhus and spotted fever. As reported previously [42], the Panbio DENV μ-capture ELISA showed a higher false positive rate than the rapid test.

Major limitations of the presented study are the relatively low case number and the strong bias to the two major DENV serotypes (DENV2, DENV3) found in the southern part of Lao PDR during the 2013 epidemics [9].

For optimal clinical and economical management and treatment of febrile disease cases in resource-poor tropical and subtropical regions where malaria and DF are endemic, patients should be tested for malaria and DENV NS1 positivity as early as possible post onset of symptoms, preferably during the first three days of illness. Even at a high rate of secondary DENV infections, early application of the fairly inexpensive NS1 rapid test will quickly identify the majority of DF cases. Only DENV NS1 negative sera from patients not suffering from malaria have to be analyzed further with the more costly DENV-specific RT-PCR and diagnostic tests targeting other pathogens.

## Supporting information

S1 Checklist STARD checklist. (PDF)

S1 Fig SD Bioline Dengue Duo test results (Colombia DF patients). Acute phase serum samples from 26 patients **(A)** with a PCR-confirmed DENV infection were analyzed with the SD Bioline Dengue Duo NS1 **(B)**, IgM **(C)** and IgG test **(D)**. Samples were stratified according to sampling day (days (d) post onset of symptoms) and Ct value in the RealStar Dengue RT-PCR. Dashed lines indicate Ct-value categories low (Ct ≤ 28.0), medium (28.0 < Ct ≤ 33.0) and high (Ct > 33.0). Open/filled circles represent samples tested negative/positive in the SD Bioline Dengue Duo test. **(E)** Filled circles represent samples tested positive in the NS1 and/or the IgM test. Sample numbers and percentages are displayed (n positive / N samples in category (percentage)).
(PDF)

S2 Fig SD Bioline Dengue Duo NS1 test results stratified according to DENV serotype (Lao PDR DF patients). Acute phase serum samples from patients infected with DENV1 (N = 2, **A-D**), DENV2 (N = 28, **E—H**), DENV3 (N = 50, **I—L**), and DENV4 (N = 12, **M—P**) were analyzed with the SD Bioline Dengue Duo NS1, IgM, and IgG test. Samples were stratified according to sampling day (days (d) post onset of symptoms) and Ct value in the RealStar Dengue RT-PCR. Dashed lines indicate Ct-value categories low (Ct ≤ 28.0), medium (28.0 < Ct ≤ 33.0) and high (Ct > 33.0). Open/filled circles represent samples tested negative/positive in the SD Bioline Dengue Duo test. **(Q)** Tabular representation and statistical analysis of subgroup characteristics and SD Bioline Dengue Duo test results; ns: not significant.
(PDF)

S3 Fig SD Bioline Dengue Duo NS1 test results do not correlate with WBC and PLT (Lao PDR DF patients). WBC **(A)** and PLT counts **(B)** measured in acute phase serum samples from 92 patients with a PCR-confirmed DENV infection. Dashed lines indicate reference values. Open/filled circles represent samples tested negative/positive in the SD Bioline Dengue Duo NS1 test. **(C)** Tabular summary of results. Statistical testing was performed using Fisher’s exact test (two-sided), ns: not significant.
(PDF)

S4 Fig Commonly used immune complex dissociation methods are not compatible with the SD Bioline Dengue Duo NS1 antigen test. Serum samples with a high, medium, or low signal in the SD Bioline Dengue Duo NS1 test **(A)** were subjected to immune complex dissociation by acid treatment **(B)** or heat/EDTA **(C)**. C: control line; T: test line.
(PDF)

S5 Fig Comparison of DENV IgG IIFT and Panbio IgG ELISA results. 74 acute phase serum samples were analyzed with the in-house DENV IgG IIFT and the Panbio DENV IgG ELISA. **(A)** Open/grey/black circles represent DENV IIFT negative/positive (low titer: < 1:2,000, high titer: ≥ 1:2,000) samples. Shaded area represents index values rated as equivocal according to the manufacturer’s recommendations. **(B)** Tabular summary of results and statistical testing.
(PDF)

S6 Fig Demographics and clinical parameters for patients with febrile disease (DENV RT PCR neg or not tested, Lao PDR, 2013–2015). **(A)** Demographics of 173 Laotian patients with febrile disease admitted to Savannakhet Provincial Hospital (2013–2015). **(B)** WBC counts. **(C)** PLT counts. Samples from patients with a confirmed diagnosis of malaria or presumptive DENV infection are indicated by filled circles and red diamonds, respectively.
(PDF)

S1 Flowchart STARD flowchart. (PDF)
