# Supplementary material for: Assessment of diagnostic and analytic performance of the SD Bioline Dengue Duo test for dengue virus (DENV) infections in an endemic area (Savannakhet province, Lao People's Democratic Republic)
Source: PLoS One. 2020 Mar 17;15(3):e0230337. doi: 10.1371/journal.pone.0230337 (PMC7077838; doi:10.1371/journal.pone.0230337)

Supporting Figure S1

A

**patients, n**	26
**age in years, median (range)**	18 (12 – 61)
**male/female gender, n (%)**	15/11 (57.7/42.3)
**dpo, median (range)**	4 (2 – 8)
**Ct, median (range)**	30.5 (22.9 – 37.0)
**Dengue serotype 1/2/3/4, n (%)**	13/7/2/4 (50.0/26.9/7.7/15.4)

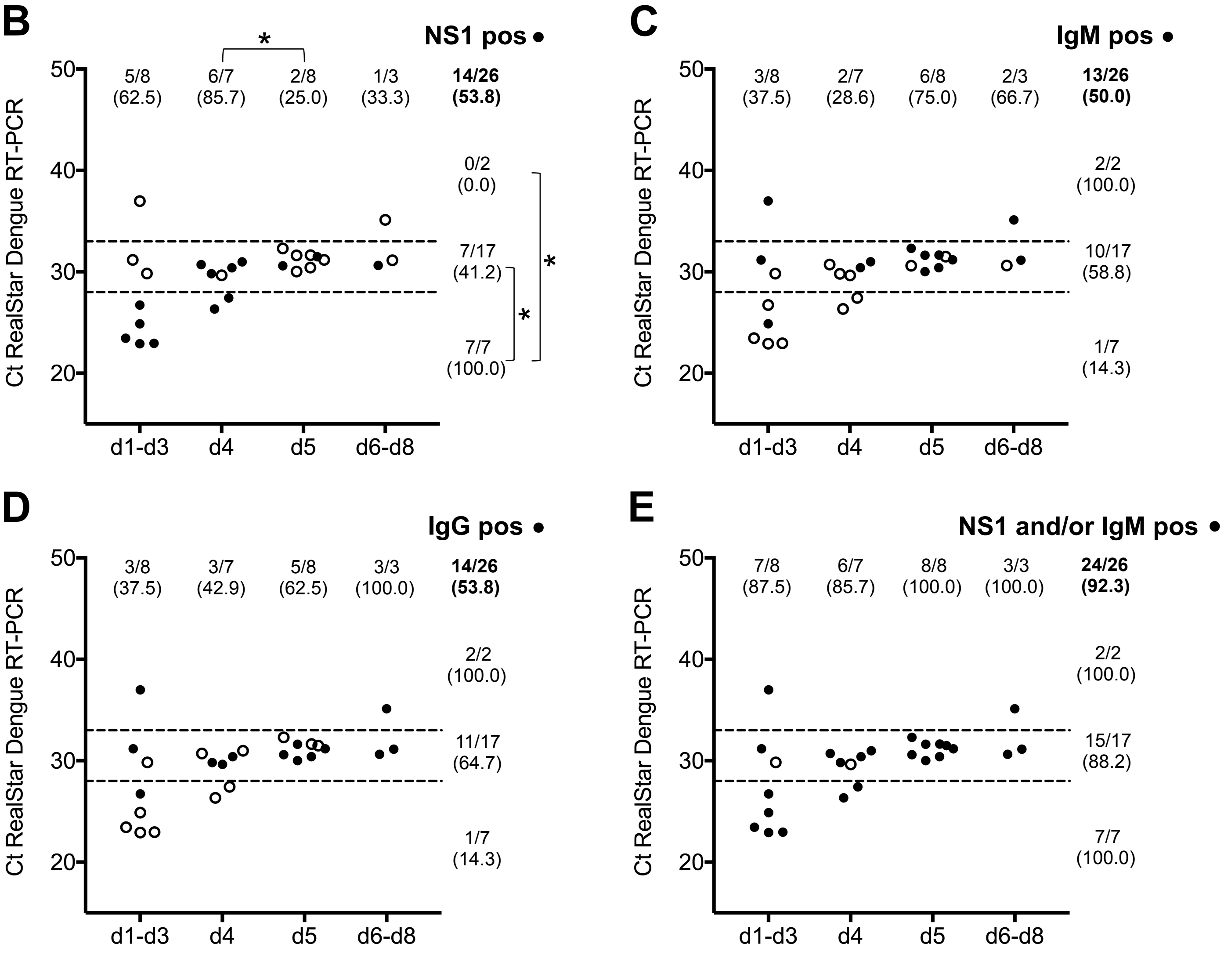

Supplement: S1 Fig — Acute phase serum samples from 26 patients (A) with a PCR-confirmed DENV infection were analyzed with the SD Bioline Dengue Duo NS1 (B), IgM (C) and IgG test (D). Samples were stratified according to sampling day (days (d) post onset of symptoms) and Ct value in the RealStar Dengue RT-PCR. Dashed lines indicate Ct-value categories low (Ct ≤ 28.0), medium (28.0 < Ct ≤ 33.0) and high (Ct > 33.0). Open/filled circles represent samples tested negative/positive in the SD Bioline Dengue Duo test. (E) Filled circles represent samples tested positive in the NS1 and/or the IgM test. Sample numbers and percentages are displayed (n positive / N samples in category (percentage)). (PDF) [file pone.0230337.s002.pdf]
