# Supplementary material for: Assessment of diagnostic and analytic performance of the SD Bioline Dengue Duo test for dengue virus (DENV) infections in an endemic area (Savannakhet province, Lao People's Democratic Republic)
Source: PLoS One. 2020 Mar 17;15(3):e0230337. doi: 10.1371/journal.pone.0230337 (PMC7077838; doi:10.1371/journal.pone.0230337)
Supplement: S2 Fig — Acute phase serum samples from patients infected with DENV1 (N = 2, A-D), DENV2 (N = 28, E—H), DENV3 (N = 50, I—L), and DENV4 (N = 12, M—P) were analyzed with the SD Bioline Dengue Duo NS1, IgM, and IgG test. Samples were stratified according to sampling day (days (d) post onset of symptoms) and Ct value in the RealStar Dengue RT-PCR. Dashed lines indicate Ct-value categories low (Ct ≤ 28.0), medium (28.0 < Ct ≤ 33.0) and high (Ct > 33.0). Open/filled circles represent samples tested negative/positive in the SD Bioline Dengue Duo test. (Q) Tabular representation and statistical analysis of subgroup characteristics and SD Bioline Dengue Duo test results; ns: not significant. (PDF) [file pone.0230337.s003.pdf]

Supporting Figure S2

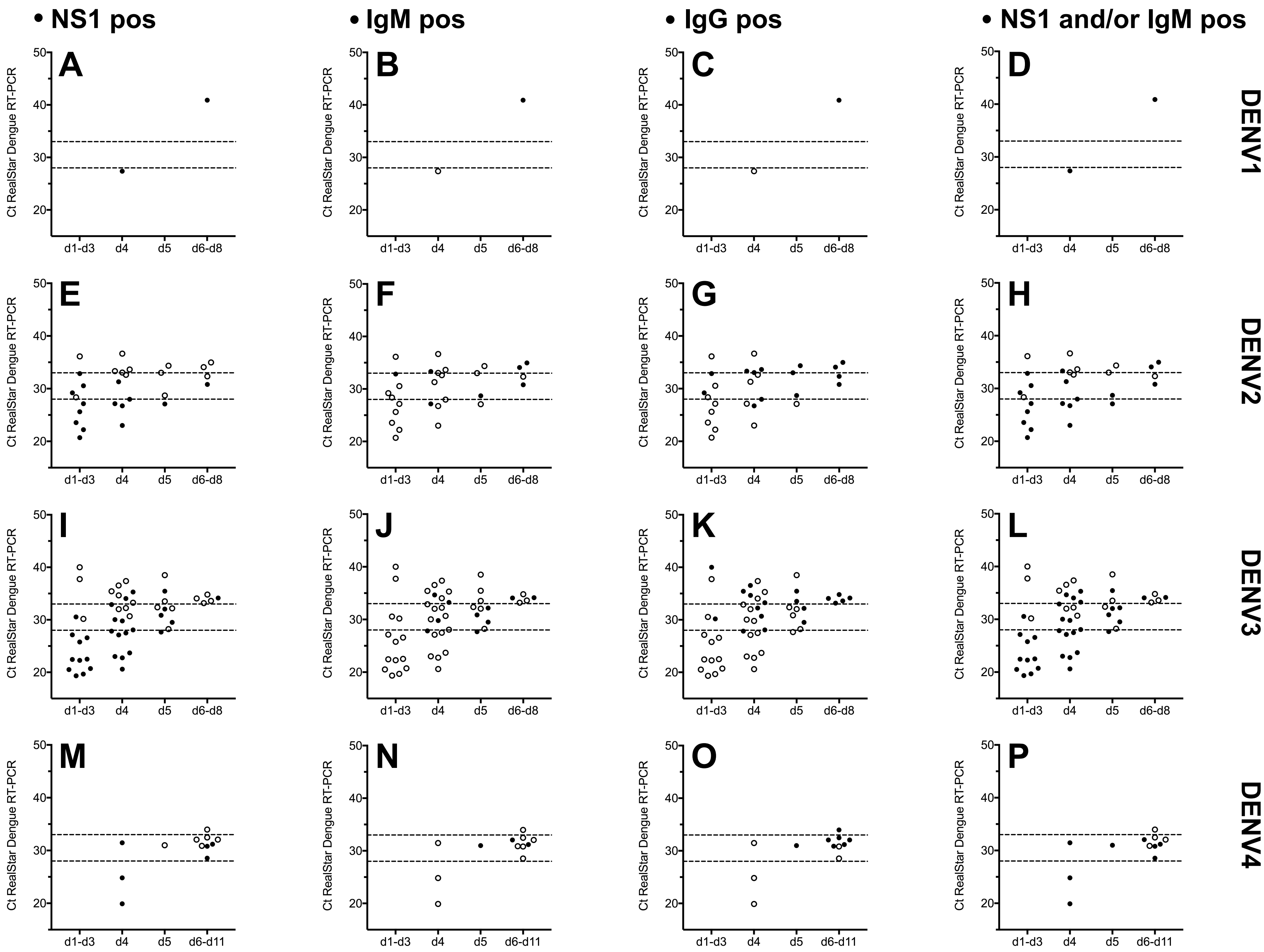

Q

	DENV1	DENV2	DENV3	DENV4	statistical testing (p)		
**# patients, n (% of all 92 patients)**	2 (2.2)	28 (30.4)	50 (54.3)	12 (13.0)	DENV2/DENV3	DENV2/DENV4	DENV3/DENV4
**age, median (range)**	19.5 (19 – 20)	20.5 (15 – 55)	21 (15 – 55)	23.5 (15 – 42)	0.9081 (ns)	0.9058 (ns)	0.8283 (ns)
**male/female gender, n (%)**	1/1 (50.0/50.0)	15/13 (53.6/46.4)	25/25 (50/50)	8/4 (66.7/33.3)	0.8162 (ns)	0.5048 (ns)	0.3501 (ns)
**dpo, median (range)**	5.5 (4 – 7)	4 (2 – 7)	4 (1 – 8)	6 (4 – 11)	0.9153 (ns)	0.0006 (***)	0.0001 (***)
**Ct, median (range)**	34.1 (27.4 – 40.9)	30.7 (20.7 – 36.7)	30.6 (19.3 – 40.0)	31.1 (19.9 – 34.0)	0.9388 (ns)	0.9822 (ns)	0.9406 (ns)
**SD Bioline Dengue Duo NS1 pos, n (%)**	2 (100.0)	15 (53.6)	30 (60.0)	6 (50.0)	0.6373 (ns)	1.000 (ns)	0.5364 (ns)
**SD Bioline Dengue Duo IgM pos, n (%)**	1 (50.0)	7 (25.0)	10 (20.0)	3 (25.0)	0.7756 (ns)	1.000 (ns)	0.7033 (ns)
**SD Bioline Dengue Duo IgG pos, n (%)**	1 (50.0)	14 (50.0)	19 (38.0)	7 (58.3)	0.3454 (ns)	0.7365 (ns)	0.3288 (ns)
**SD Bioline Dengue Duo NS1 or IgM pos, n (%)**	2 (100.0)	19 (67.9)	34 (68.0)	8 (66.7)	1.0000 (ns)	1.000 (ns)	1.000 (ns)
