# Supplementary material for: Assessment of diagnostic and analytic performance of the SD Bioline Dengue Duo test for dengue virus (DENV) infections in an endemic area (Savannakhet province, Lao People's Democratic Republic)
Source: PLoS One. 2020 Mar 17;15(3):e0230337. doi: 10.1371/journal.pone.0230337 (PMC7077838; doi:10.1371/journal.pone.0230337)
Supplement: S3 Fig — WBC (A) and PLT counts (B) measured in acute phase serum samples from 92 patients with a PCR-confirmed DENV infection. Dashed lines indicate reference values. Open/filled circles represent samples tested negative/positive in the SD Bioline Dengue Duo NS1 test. (C) Tabular summary of results. Statistical testing was performed using Fisher’s exact test (two-sided), ns: not significant. (PDF) [file pone.0230337.s004.pdf]

Supporting Figure S3 (revised version)

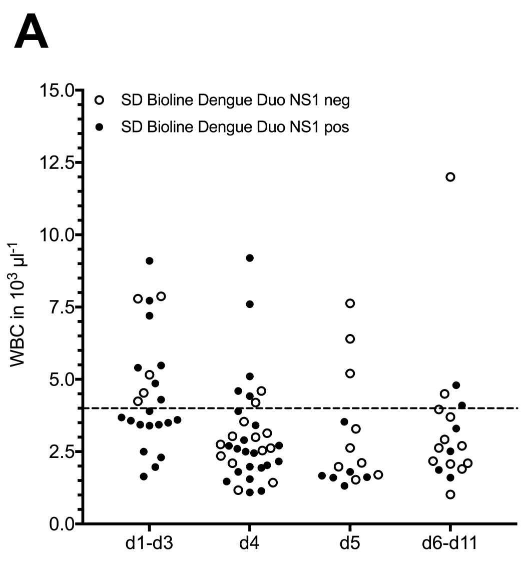

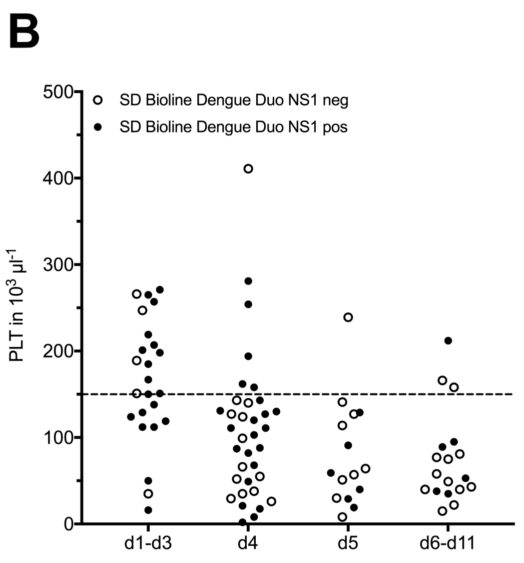

C

category	patients, n (%)	dpo, median (range)	Ct, median (range)	SD Bioline NS1 pos, n (%)	result statistical testing
WBC < 4.0 x 10^3 µl^{-1}	66 (71.7)	4 (2 – 11)	30.9 (19.3 – 37.4)	39 (59.1)	p = 0.6485 (ns)
WBC ≥ 4.0 x 10^3 µl^{-1}	26 (28.3)	4 (1 – 7)	30.1 (19.7 – 40.9)	14 (53.8)	
PLT < 150.0 x 10^3 µl^{-1}	67 (72.8)	4 (2 – 11)	31.2 (19.3 – 40.0)	36 (53.7)	p = 0.2447 (ns)
PLT ≥ 150.0 x 10^3 µl^{-1}	25 (27.2)	3 (1 – 7)	29.8 (19.7 – 40.9)	17 (68.0)	
