# Supplementary figures and images for: Assessment of diagnostic and analytic performance of the SD Bioline Dengue Duo test for dengue virus (DENV) infections in an endemic area (Savannakhet province, Lao People's Democratic Republic)
Source: PLoS One. 2020 Mar 17;15(3):e0230337. doi: 10.1371/journal.pone.0230337 (PMC7077838; doi:10.1371/journal.pone.0230337)

## Supporting Figure S4

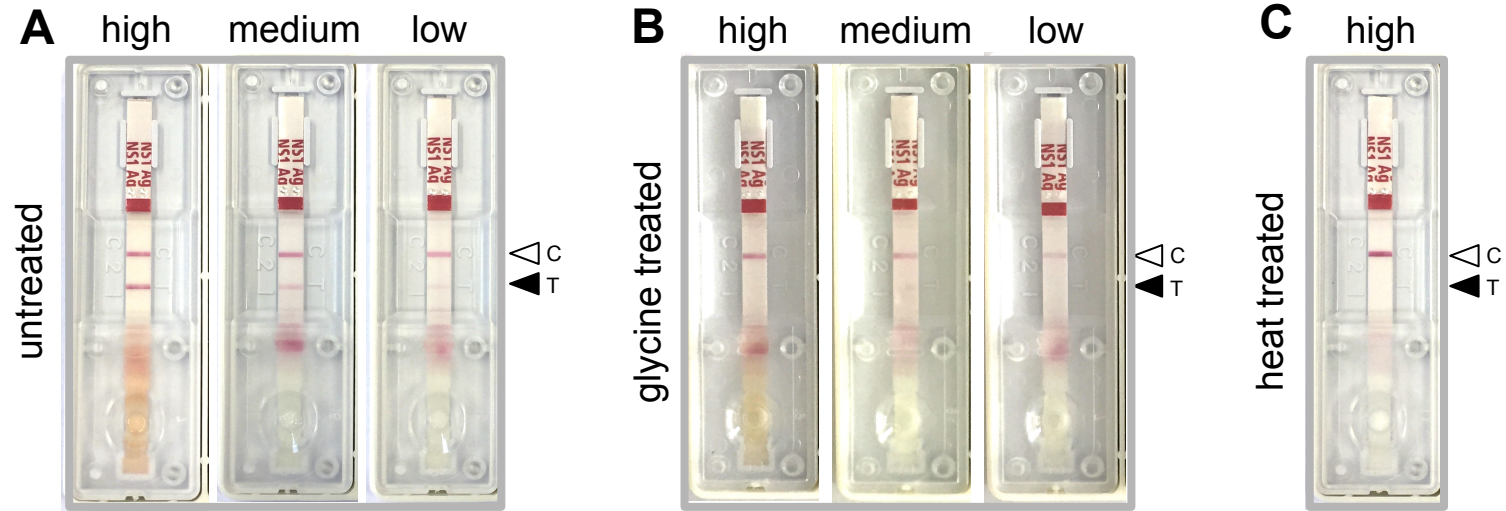

Supplement: S4 Fig — Serum samples with a high, medium, or low signal in the SD Bioline Dengue Duo NS1 test (A) were subjected to immune complex dissociation by acid treatment (B) or heat/EDTA (C). C: control line; T: test line. (PDF) [file pone.0230337.s005.pdf]

# STARD Flow Chart (serum samples Lao PDR)

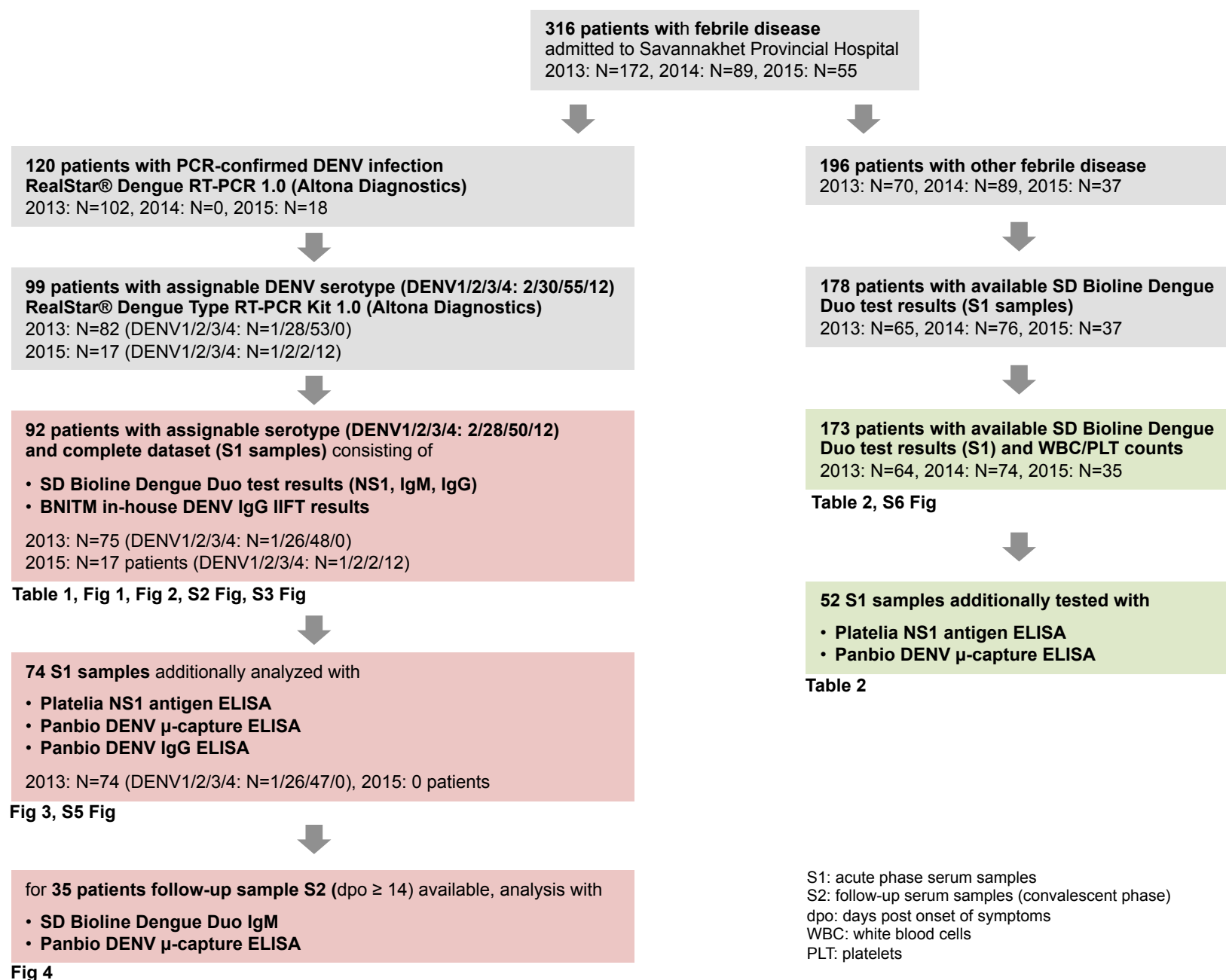

# STARD Flow Chart (serum samples Colombia)

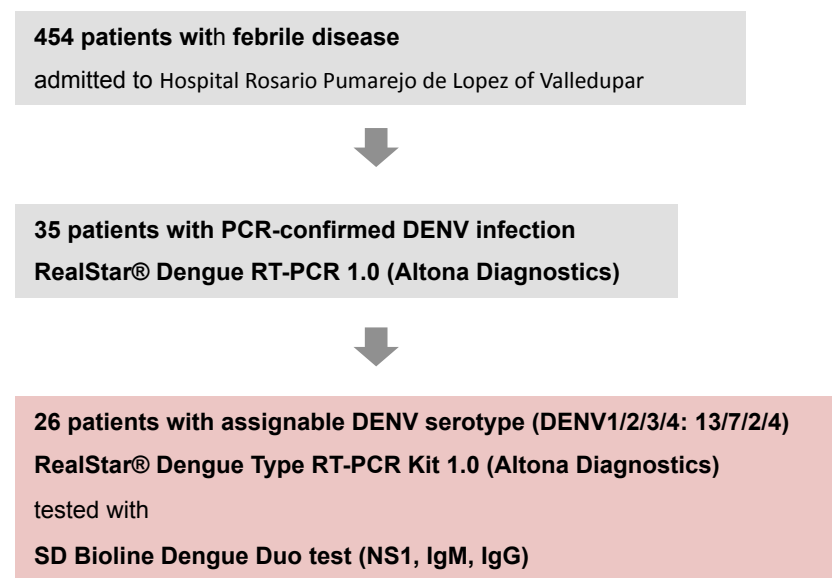

S1 Fig

Supplement: S1 Flowchart — (PDF) [file pone.0230337.s008.pdf]
