# Supplementary material for: Assessment of diagnostic and analytic performance of the SD Bioline Dengue Duo test for dengue virus (DENV) infections in an endemic area (Savannakhet province, Lao People's Democratic Republic)
Source: PLoS One. 2020 Mar 17;15(3):e0230337. doi: 10.1371/journal.pone.0230337 (PMC7077838; doi:10.1371/journal.pone.0230337)
Supplement: S5 Fig — 74 acute phase serum samples were analyzed with the in-house DENV IgG IIFT and the Panbio DENV IgG ELISA. (A) Open/grey/black circles represent DENV IIFT negative/positive (low titer: < 1:2,000, high titer: ≥ 1:2,000) samples. Shaded area represents index values rated as equivocal according to the manufacturer’s recommendations. (B) Tabular summary of results and statistical testing. (PDF) [file pone.0230337.s006.pdf]

Supporting Figure S5

A

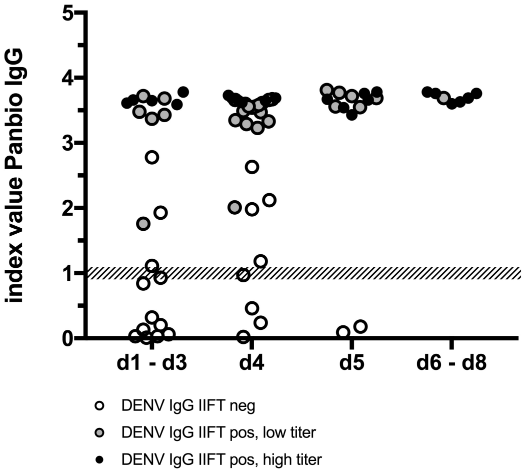

B

day post onset of symptoms	n (%)	IgG pos (n (%)) in	
		**DENV IgG IIFT**	**Panbio DENV IgG ELISA**
d1 – d3	23 (31.1)	11 (47.8)	14 (60.9)
d4	30 (40.5)	22 (73.3)	26 (86.7)
d5	14 (18.9)	12 (85.7)	12 (85.7)
d6 – d8	7 (9.5)	7 (100.0)	7 (100.0)
all samples	74 (100.0)	52 (70.3)	59 (79.7)
% agreement		90.5	
Cohen' kappa		0.75	
