# Supplementary material for: Assessment of diagnostic and analytic performance of the SD Bioline Dengue Duo test for dengue virus (DENV) infections in an endemic area (Savannakhet province, Lao People's Democratic Republic)
Source: PLoS One. 2020 Mar 17;15(3):e0230337. doi: 10.1371/journal.pone.0230337 (PMC7077838; doi:10.1371/journal.pone.0230337)

Supporting Figure S6

A

**patients, n**	173
**age in years, median (range)**	31 (15 - 65)
**male/female gender, n (%)**	106/67 (61.3/38.7)
**dpo, median (range)**	5 (1 – 30)
**WBC in 10^3 μl^{-1}, median (range)**	6.2 (1.6 – 19.0)
**WBC < reference, n (%)**	49 (28.3)
**PLT in 10^3 μl^{-1}, median (range)**	203.0 (6.4 – 572.0)
**PLT < reference, n (%)**	49 (28.3)

B

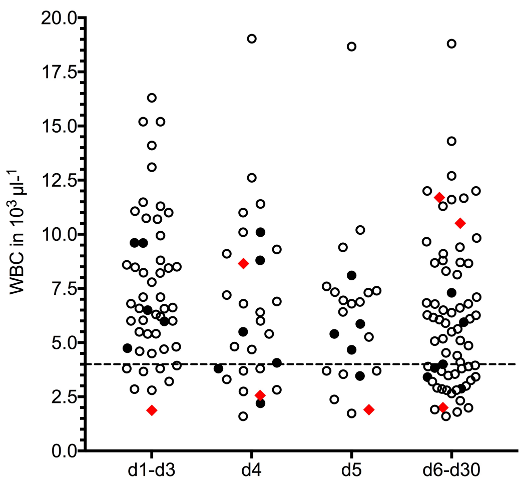

C

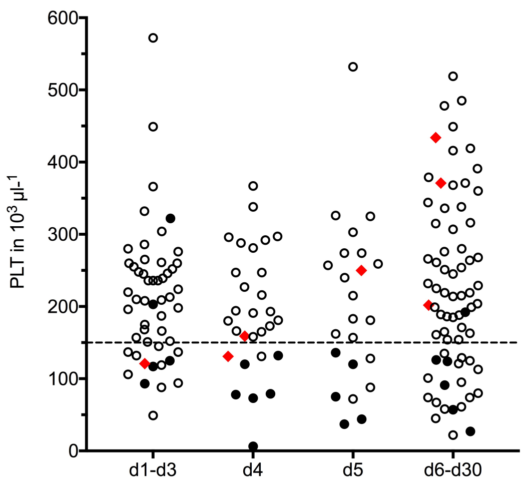

Supplement: S6 Fig — (A) Demographics of 173 Laotian patients with febrile disease admitted to Savannakhet Provincial Hospital (2013–2015). (B) WBC counts. (C) PLT counts. Samples from patients with a confirmed diagnosis of malaria or presumptive DENV infection are indicated by filled circles and red diamonds, respectively. (PDF) [file pone.0230337.s007.pdf]
